# Complete Genome Sequence of Mycobacterium tuberculosis DKC2, the Predominant Danish Outbreak Strain

**DOI:** 10.1128/MRA.01554-18

**Published:** 2019-01-24

**Authors:** Anders Norman, Dorte Bek Folkvardsen, Søren Overballe-Petersen, Troels Lillebaek

**Affiliations:** a International Reference Laboratory of Mycobacteriology, Statens Serum Institut, Copenhagen, Denmark; b Department of Bacteria, Parasites and Fungi, Statens Serum Institut, Copenhagen, Denmark; University of Maryland School of Medicine

## Abstract

The largest clonal outbreak of Mycobacterium tuberculosis infection in Scandinavia has been monitored by the International Reference Laboratory of Mycobacteriology (IRLM) since 1992. Here, we present the complete genome sequence of M. tuberculosis strain DKC2 substrain PP1, a representative isolate collected in 1993 from a Danish patient with pulmonary tuberculosis.

## ANNOUNCEMENT

Worldwide, tuberculosis (TB) is the most lethal infectious disease, with 1.6 million deaths in 2017. An estimated one-fourth of the world’s population is infected with its causative agent, Mycobacterium tuberculosis, with a 5 to 10% chance in their lifetime of developing active disease. The Danish “Cluster 2/1112-15” (DKC2) TB outbreak is the largest of its kind in Scandinavia, having accounted for more than 1,000 cases in Denmark and Greenland since 1992 (1–3). The DKC2 genotype belongs to the M. tuberculosis complex lineage 4 and is closely related to the reference strain H37Rv, which belongs to sublineage 4.10/PGGE3 (4). The isolate DKC2-PP1 was grown on Middlebrook 7H10 agar, and genomic DNA was purified via the cetyltrimethylammonium bromide (CTAB) method (5). Whole-genome sequencing was performed on the MiSeq Illumina and Oxford Nanopore Technologies (ONT) MinION Mk1B platforms. A paired-end (2 × 150-bp) MiSeq library was prepared with the Nextera XT kit. Two barcoded MinION libraries were prepared using the Ligation Sequencing 1D and Native Barcoding 1D kits, respectively, and sequenced on R9.4 SpotON flow cells. MinION base calling was performed with ONT’s Albacore 2.1.3 software, which yielded 902,071 reads. Sequencing adaptors were removed with Porechop 0.2.2 (https://github.com/rrwick/Porechop), resulting in 1.29 gigabases (Gb) with an *N*_50_ read length of 2.0 kb. Before *de novo* assembly, reads shorter than 1 kb were removed, resulting in 1 Gb with an *N*_50_ read length of 2.6 kb. Unicycler 0.4.0 (6) was used to assemble long reads, yielding 2 unitigs of 4.37 Mb and 66.3 kb, respectively. The assembly was finalized in Geneious 9.1.8 by joining the four overlapping ends of the 2 unitigs, with H37Rv (GenBank accession no. NC_000962) as a guide, to construct a 4,405,921-bp circular scaffold with 225× ONT coverage. Then, 2,180,077 MiSeq read pairs (2 × 314 Mb), supplemented with reads from the DKC2 outbreak deposited to the European Nucleotide Archive (ENA) under project no. PRJEB20214, were used to polish the genome repeatedly until no variants could be detected.

The finished sequence consists of a single circular 4,409,544-bp chromosome with a GC content of 65.6%. The initial ONT-only scaffold was 99.8% accurate compared to this sequence. Automated genome annotation was performed with Prokka 1.13 (7) and curated in Geneious, yielding 4,175 genes consisting of 4,052 coding regions (CDS), 53 tRNAs, 43 noncoding RNAs (ncRNAs) (CRISPR spacers), 35 putatively regulatory RNAs, and 3 rRNAs. Mobile insertion sequence elements were identified using the ISfinder server (https://isfinder.biotoul.fr/) and by alignment to H37Rv. Notable genome differences between this sequence and that of H37Rv include the presence of an inverted RvD2 region, small (<20-bp) frameshifts or truncations to Rv0090, Rv0402c, Rv0456c, Rv1320c, Rv1900c, Rv1933c, Rv3033, Rv3277, Rv3350c, Rv3533c, and Rv3895c, and deletion of the entire Rv1758-to-Rv1762c region. Furthermore, the ESX-2 type VII secretion system gene corresponding to Rv3894c contains a 2.3-kb deletion, which resulted in a significantly truncated (770-amino acid) version of EccC2.

### Data availability.

The complete genome sequence has been deposited in the European Nucleotide Archive (ENA) under accession no. LR027516. Raw read data from this study have been deposited under run accession no. ERR2724039, ERR2724040, and ERR2724042 under sample no. ERS1691204.
